# Purinergic signaling in the male reproductive tract

**DOI:** 10.3389/fendo.2022.1049511

**Published:** 2022-11-07

**Authors:** Larissa Berloffa Belardin, Kéliane Brochu, Christine Légaré, Maria Agustina Battistone, Sylvie Breton

**Affiliations:** ^1^ Centre Hospitalier Universitaire de Québec - Research Centre and Department of Obstetrics, Gynecology and Reproduction, Faculty of Medicine, Université Laval, Québec, QC, Canada; ^2^ Nephrology Division, Department of Medicine, Massachusetts General Hospital, Harvard Medical School, Boston, MA, United States

**Keywords:** epididymis, testis, vas deferens, ATP, adenosine, inflammation, purinergic receptors, sperm

## Abstract

Purinergic receptors are ubiquitously expressed throughout the body and they participate in the autocrine and paracrine regulation of cell function during normal physiological and pathophysiological conditions. Extracellular nucleotides activate several types of plasma membrane purinergic receptors that form three distinct families: P1 receptors are activated by adenosine, P2X receptors are activated by ATP, and P2Y receptors are activated by nucleotides including ATP, ADP, UTP, UDP, and UDP-glucose. These specific pharmacological fingerprints and the distinct intracellular signaling pathways they trigger govern a large variety of cellular responses in an organ-specific manner. As such, purinergic signaling regulates several physiological cell functions, including cell proliferation, differentiation and death, smooth muscle contraction, vasodilatation, and transepithelial transport of water, solute, and protons, as well as pathological pathways such as inflammation. While purinergic signaling was first discovered more than 90 years ago, we are just starting to understand how deleterious signals mediated through purinergic receptors may be involved in male infertility. A large fraction of male infertility remains unexplained illustrating our poor understanding of male reproductive health. Purinergic signaling plays a variety of physiological and pathophysiological roles in the male reproductive system, but our knowledge in this context remains limited. This review focuses on the distribution of purinergic receptors in the testis, epididymis, and vas deferens, and their role in the establishment and maintenance of male fertility.

## Introduction

**Figure 1:**
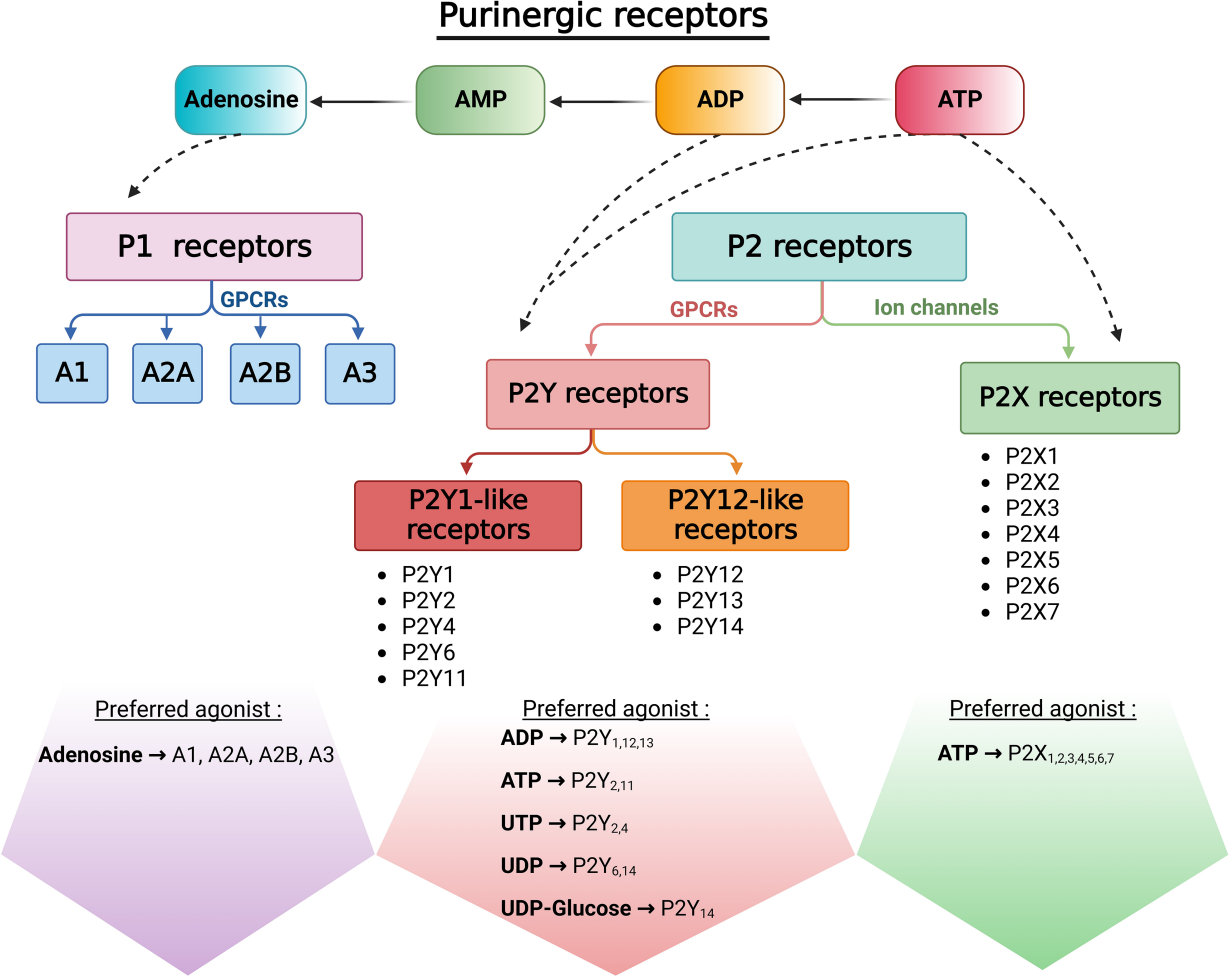
Classification of purinergic receptors. Once released into the extracellular space, ATP is rapidly hydrolyzed by ectonucleotidases to generate ADP, AMP, and adenosine. Adenosine activates P1 receptors, a family of G-protein coupled receptors (GPCRs). P2X receptors are non-selective cation channels that open in response to ATP by their only endogenous ligand. In addition to ATP and ADP, P2Y metabotropic receptors can mediate cellular responses to uracil nucleotides and nucleotides sugars.

Purinergic signaling was first described in 1929 by Drury and Szent-Gyorgyi who showed extracellular adenosine-induced transient effects on the mammalian heart (1). The term “purinergic” was then proposed by Geoffrey Burnstock who demonstrated in 1970 that adenosine 5’-triphosphate (ATP) acts as an extracellular mediator in the gut (2). It is now known that, through evolutionarily conserved autocrine and paracrine communication mechanisms, extracellular adenosine, ATP, and other types of nucleotides activate members of the membrane-bound purinoceptor family (3–8). Receptors that bind adenosine were named P1 purinergic receptors, and those that bind ATP were named P2 purinergic receptors (9) (**Figure 1**). P1 receptors are G protein-coupled receptors (GPCRs) activated by adenosine. The P2 family was later subdivided into P2X ionotropic receptors and P2Y metabotropic receptors. P2X receptors are ligand-gated non-selective cation channels activated by ATP, and P2Y receptors are GPCRs activated by adenine and several uracil nucleotides (ATP, ADP, UTP, UDP and UDP-glucose (6, 10–19) (**Figure 1**).

Purinergic receptors are ubiquitously expressed throughout the body and they participate in intercellular communication during normal physiological and pathophysiological conditions. This review focuses on the distribution of purinergic receptors in the male reproductive tract, and their role in the establishment and maintenance of male fertility.

## The purinergic receptor family

P1, P2X, and P2Y receptors are expressed by virtually all cell types, and they participate in several cell functions, including cell proliferation, differentiation, and death, through the modulation of a variety of intracellular signaling pathways. Other physiological functions regulated by these receptors include smooth muscle contraction, vasodilatation, and transepithelial transport of water, solute, and protons, as well as pathological pathways including inflammation.

**Figure 2:**
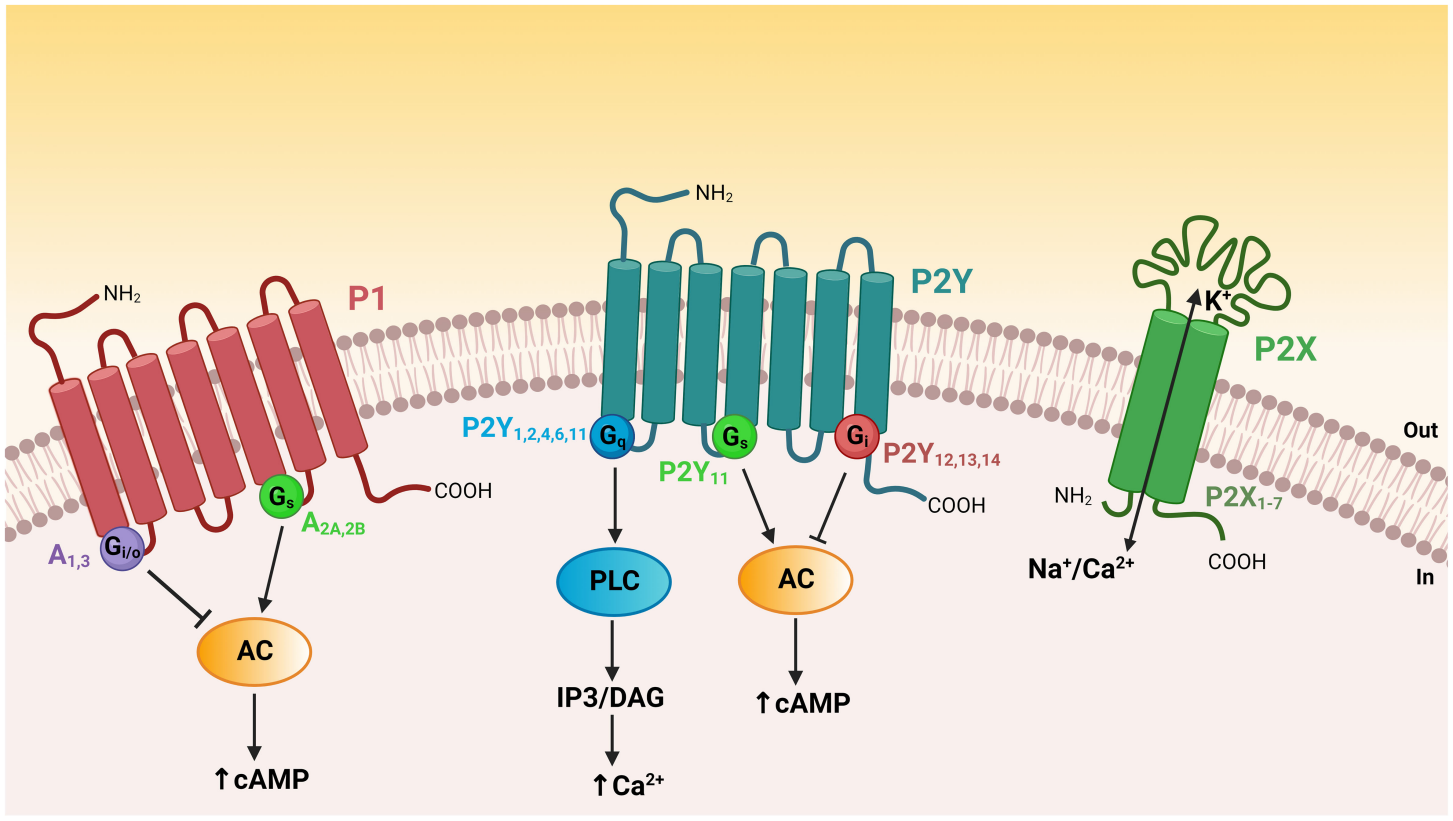
Topology of purinergic receptors and intracellular signaling pathways. The P2X receptors have two transmembrane domains with both their N- and C-termini on the cytoplasmic side of the plasma membrane. Activation of P2X receptors by extracellular ATP opens a non-selective cation channel, resulting in calcium and sodium influx or potassium efflux. P1 and P2Y receptors are GPCRs possessing seven transmembrane domains with an extracellular N-terminus domain and an intracellular C-terminus domain. P2Y1-like receptors (P2Y_1,2,4,6,11_) are coupled to G_q_ and their activation leads to phospholipase C (PLC) activation, generation of IP3 and DAG, and intracellular Ca^2+^ release. The P2Y11 receptor couples to both G_q_ and G_s_ to increase intracellular Ca^2+^ and cAMP. P2Y12-like receptors (P2Y_12,13,14_) are coupled to G_i_ and thus reduce intracellular cAMP levels through inhibition of adenylyl cyclase (AC). Adenosine P1 receptors differentially regulate the intracellular concentration of cAMP. A1 and A3 receptors are coupled to G_i/o_ and induce a diminution in cAMP levels, whereas A2A and A2B receptors are coupled to G_s_ and their activation leads to an elevation in intracellular cAMP.

The P2X receptor family includes seven homotrimeric (P2X1–7) and several heterotrimeric isoforms (e.g. P2X2, 4, 6) (**Figure 2**). These isoforms can be distinguished through their ligand affinities, activation and desensitization kinetics, as well as specific pharmacological fingerprints (11, 20). P2X receptors are formed by two transmembrane domains separated by a large extracellular domain and have both their N- and C-termini domains located on the cytoplasmic side of the plasma membrane (21). Activation of P2X receptors by ATP triggers conformational changes that lead to the influx of calcium and sodium, or the efflux of potassium *via* the opening of a non-selective cation-permeable channel pore (22). The P2Y receptors consist of seven transmembrane domains with an extracellular N-terminus domain and intracellular C-terminus domain (**Figure 2**). This family is formed by eight isoforms (P2Y1,2,4,6,11,12,13,14) that are coupled to different G proteins and activate distinct intracellular signaling pathways (11, 21). The missing numbers in the P2Y receptor series indicate receptors that are unresponsive to nucleotides. The gene coding for the P2Y11 receptor is absent in the rodent genome. The P2Y1-like receptors P2Y1,2,4,6 activate G_q_ and phospholipase C (PLC). PLC hydrolyzes phosphatidylinositol-4,5-bisphosphate to form inositol1,4,5-trisphosphate (IP3) and diacylglycerol (DAG), leading to the release of Ca^2+^ from intracellular calcium storage organelles and activation of protein kinase C (PKC), respectively. The P2Y11 receptor couples to both G_q_ and G_s_, and its activation thus increases intracellular Ca^2+^ and cAMP. The P2Y12-like receptors, P2Y12,13,14 are coupled to G_i_ and reduce intracellular cAMP concentration through inhibition of adenylyl cyclase (**Figure 2**). P2Y receptors were later shown to modulate additional intracellular signaling pathways, including the recruitment of the G_βγ_ subunit followed by activation of phosphatidylinositol-4,5-biphosphate 3-kinase (PI3K), PLC-β2 and PLC-β3, GPCR kinase 2 and 3, Rho, and MAPKs (23). Different affinities of the P2X and P2Y receptors for ATP and nucleotides confer functional specificity and physiological flexibility to various cellular functions. As such, the P2 receptor expression profile of a given cell underlies its unique response phenotype upon ATP stimulation. In addition, the degradation of extracellular ATP into ADP or UDP modulates the cellular response *via* activation of different P2X and P2Y receptors (24, 25). On the other hand, ATP is rapidly hydrolyzed into adenosine by extracellular ectonucleotidases (26–29). Adenosine binds to P1 receptors and triggers opposite effects in cells by either inducing a decrease in intracellular cAMP, through ADORA1 (A1) and ADORA3 (A3) receptors that are coupled to G_i/o_, or a cAMP increase through ADORA2A (A2A) or ADORA2B (A2B) receptors that are coupled to G_s_ (**Figure 2**).

## Physiological and pathophysiological roles of purinergic receptors

Extracellular nucleotides are released in the extracellular fluid during cell lysis, exocytosis, or by efflux through transport proteins located in the cell membranes (30). These nucleotides act in an autocrine or paracrine manner to regulate cellular activity in physiological and pathophysiological conditions *via* purinergic signaling. Adenosine has been administered clinically since the 1940s to treat supraventricular tachycardia (31, 32), and P2Y12 antagonists are widely used as anti-thrombotic agents (19). In general, adenosine is considered to be tissue protective and anti-inflammatory, in contrast to ATP and UDP-glucose, which are damage-associated molecular pattern (DAMP) molecules that have pro-inflammatory effects (31, 33, 34). In trauma, ischemia, stroke, etc., high levels of extracellular ATP are related to massive inflammatory response and cell death (35). Activation of P2Y1, P2Y2, P2Y6, P2X1 and P2X7 triggers the production of chemokines in monocytes and dendritic cells, and promotes the recruitment of inflammatory cells to inflamed areas (36, 37). Tissue-resident macrophages express P2X4, which mediates Ca^2+^-dependent PLA2 and COX signaling (36). In addition, ATP acts as a “find-me” signal to promote the effective clearance of apoptotic cells by phagocytes through activation of P2Y2 (38). ATP and adenosine drive neutrophil migration through P2X1, P2Y2 and A3 receptors in an autocrine manner (37, 39).

Several other purinergic modulators are now being considered to treat central nervous system (CNS), kidney, heart, and lung diseases, as well as diabetes, gouty arthritis, obesity, and cancer (6, 19, 31, 33, 34, 40–47). In the CNS, P2X and P2Y receptors regulate synaptic transmission (48). The P2Y13 receptor, for example, participates in pain transmission and neuroprotection (49). P2X3,4,7 and P2Y12 and P2Y14 are involved in maladaptive pain neuroplasticity and their antagonists have been proposed as therapeutic agents to reduce chronic or inflammatory neuropathic pain (44, 50). P2Y1 and P2Y13 are involved in neuronal differentiation and axonal elongation (51, 52), and the participation of P2Y1 is down-regulated by the activation of P2Y13 by ADP or the activation of P2X7 (52). During neurogenesis and early brain development, ectonucleotidases can lead to the downregulation of purinergic signaling, which controls progenitor cell growth and helps neuronal differentiation (53). P2Y12 located in microglial cells directs the extension of microglial processes and participates in the rapid closure of the blood-brain barrier following CNS injury (54). Microglial P2Y12 can also participate in neuron-glia intercellular communication following its activation by ATP secreted by neurons. This pathway further contributes to providing a neuroprotective mechanism after acute brain injury (55). The neurological action of purinergic receptors is important to male reproductive function, where neuro-muscular interactions play a key role in muscle contractions in the vas deferens and epididymis (discussed below). In addition, ATP participates in penile erection, through its action in the corpus cavernosum (56–58).

Purinergic receptors also participate in the regulation of the cardiovascular system (59). In the heart, adenosine, acting mainly on the A1 receptor, influences the cardiac pacemakers causing a negative chronotropic effect (60). In addition, following a decrease in oxygen concentration, erythrocytes release ATP that binds P2Y1 and P2Y2 receptors in blood vessels, leading to an increase in intracellular Ca^2+^ and production of nitric oxide (NO) that results in vasodilatation (61, 62). In the testis, purinergic signaling is also involved in circulatory disorders through an increase in NO production in spermatic veins and seminal plasma in patients with varicocele (63).

In addition, luminal nucleotides and adenosine are potent modulators of transepithelial transport in nearly all tubular organs. Several P2X, P2Y, and all P1 receptors are differentially expressed throughout the kidney and contribute to the regulation of renal function (33). A particularity of several purinergic receptors is their apical localization in epithelial cells (4). ATP release is activated by mechanical stimulation such as cell swelling and shear stress, and ATP together with its hydrolysis product adenosine are part of local intrarenal mechanisms that help regulate glomerular filtration rate (GFR) (64, 65), and water and electrolyte transport in collecting duct principal cells (33, 66). Renal disease is often associated with acidosis and intra-renal adenosine has a protective effect following kidney injury (43, 67–69). Extracellular adenosine stimulates proton secretion in the collecting duct, *via* activation of A2A and A2B receptors located on the apical membrane of intercalated cells (70). This would contribute to reducing acidosis, thus providing additional evidence of the protective effect of adenosine in the kidney. In contrast, activation of P2Y14 by UDP-glucose triggers sterile inflammation in the kidney, through the production of pro-inflammatory chemokines in intercalated cells, and infiltration of neutrophils and monocytes, which contribute to renal tubule damage following renal ischemia-reperfusion injury (45). ATP can also have a pro-inflammatory role in the kidney *via* activation of P2X7. While this receptor is barely detectable in healthy adult kidneys, its expression is increased in rodent models of kidney disease (33). Interestingly, P2X7 KO mice do not develop kidney fibrosis following ureteric obstruction (71).

Activation of purinergic receptors by ATP is now proposed to be part of a non-TLR4-mediated mechanism that protects against bacterial infections in the urinary tract and kidney (33). Bacterial infections can induce host- or bacteria-related ATP local signaling. Exposure of human uroepithelial A498 cells to *Escherichia coli* (*E. coli*) induces the release of ATP and P2Y12 activation leading to IL-8 release. Interestingly, the *E. coli* toxin α-hemolysin (HlyA), forms a large ATP pore in the infected cells that contributes to the inflammatory response (72). HlyA is a stronger inducer of IL-1 production compared to lipopolysaccharide (LPS) (72).

P2X7 is one of the best characterized purinergic receptors in the context of cancer (73), including ovarian (74), mesothelioma (75), pancreatic cell (76), osteosarcoma cell (77), endometrial (78) and skin cancer (79, 80). Activation of P2X7 by ATP leads to a decrease in intracellular potassium levels, activation of inflammasomes followed by cell death by necrosis or apoptosis, and mediates an inflammatory response by causing the release of interleukin IL-1β and IL-18 (33, 36, 72, 81). This pathway confers an anti-tumor role of P2X7 that contributes to the immunogenic death of tumor cells. In addition, P2Y2 was shown to inhibit cell proliferation in several cancers, such as human colorectal carcinoma (82), human oesophageal cancer (83), nasopharyngeal carcinoma (84), endometrial carcinoma (85). Some antitumor therapies, such as chemotherapy and radiation therapy, induce tissue damage and cell death, leading to the release of many DAMP molecules – including ATP (86). These nucleotides in the tumor microenvironment serve as the interface of interaction with the immune system (87).

In the respiratory system, ATP participates in the secretion of mucin through activation of P2Y2 (88) and it regulates the respiratory rhythm (89). UDP-glucose acting on P2Y14 is involved in airway eosinophilia associated with asthma (46). In that study, mice treated with P2Y14 antagonists as well as mice lacking P2Y14 had decreased eosinophilia and airway hyperresponsiveness indicating that this receptor could be targeted to treat asthma exacerbations and glucocorticoid-resistant forms of asthma. P2Y12 also plays a role in bronchial asthma (90), where it is involved in the migration of platelets to the lung tissue and their subsequent activation following allergen exposure (91, 92).

## Purinergic receptors in the male reproductive tract and maturing spermatozoa

Purinergic regulation of male reproductive organs was first described more than 20 years ago in studies showing the physiological action of extracellular ATP and adenosine in the vas deferens (93, 94). Years later, the role of P2X receptors in fertility gained particular interest when P2X1 knockout mice were proven infertile, due to a reduction in vas deferens contractions leading to impaired sperm delivery in the ejaculate (95, 96). An increasing number of purinergic receptors are now being described all along the male reproductive tract. Here we focus on the role of purinergic signaling in selected male organs, including the testis, the epididymis, and vas deferens, as well as in spermatozoa. Readers interested in organs located more distally in the male reproductive tract are referred to other excellent reviews (97–99).

### Testis

The testis is composed of seminiferous tubules - where spermatogenesis takes place – that are surrounded by an interstitial space that includes various cell types such as Leydig cells and peritubular myoid cells. Sertoli cells in the seminiferous tubules are responsible for the differentiation of germ cells located at the base of the tubule. These maturing cells migrate along the side of Sertoli cells and become spermatozoa, which are delivered into the tubule lumen (100–103).

**Figure 3:**
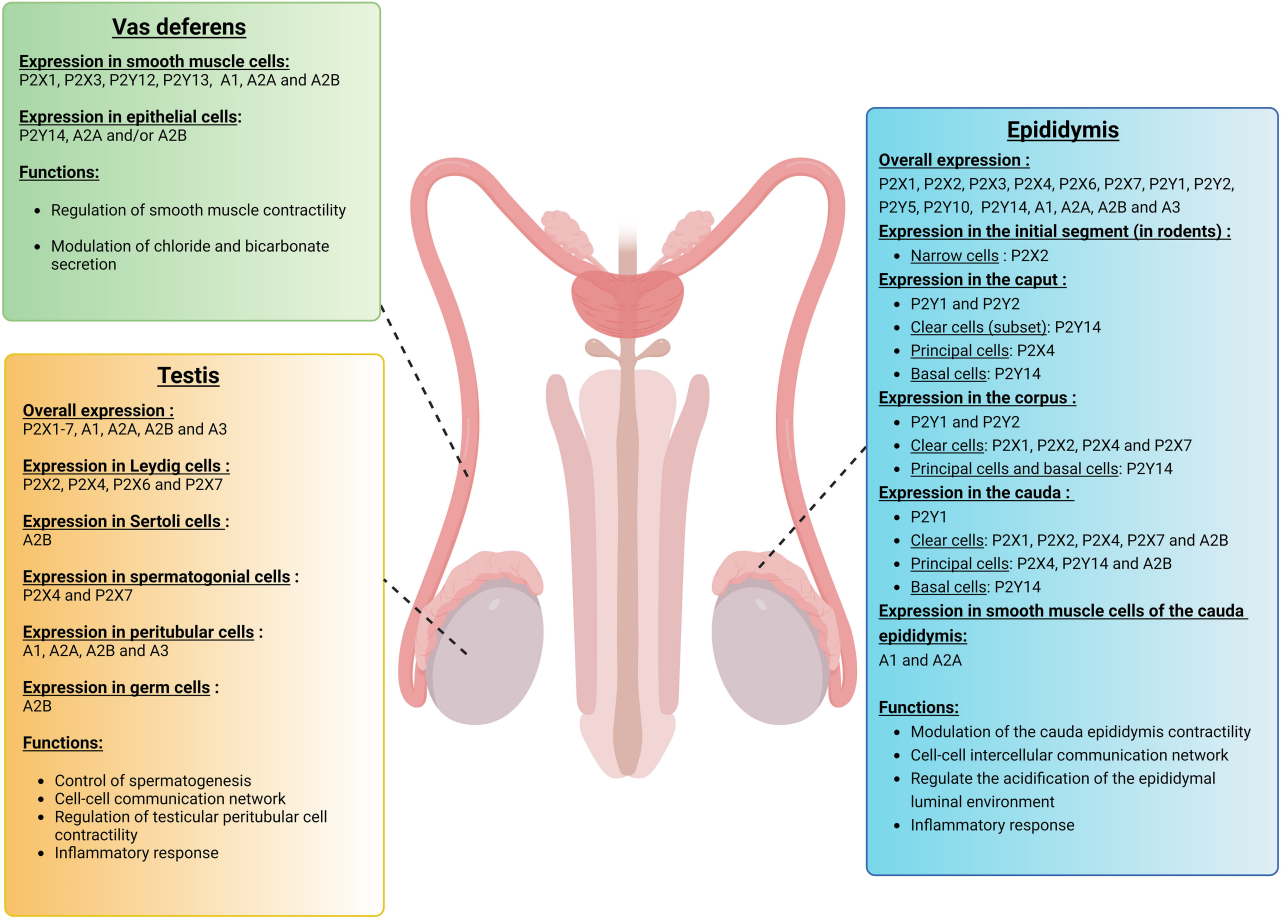
Expression of purinergic receptors in vas deferens, testis, and epididymis. Different sets of purinergic receptors belonging to the P1, P2X, and P2Y families are expressed in the vas deferens, testis, and epididymis. These receptors are involved in various cell functions, including smooth muscle contraction, vasodilatation, transepithelial transport as well as inflammation, and thus play a role in the establishment of male fertility. In the vas deferens, purinergic signaling mostly participates in the regulation of smooth muscle contraction and quality of the semen. In the testis, various purinergic receptors are expressed in a cell-specific manner, including Leydig cells, Sertoli cells, spermatogonial cells, testicular peritubular cells, and germ cells. These purinergic receptors play a critical role in testicular autocrine and paracrine communication. In the epididymis, different subtypes of purinergic receptors are expressed in a cell-specific and segment-specific manner. Purinergic signaling in the epididymis is part of a complex intercellular communication network that leads to the establishment of an optimal acidic extracellular environment.

Purinergic signaling is a critical component of testicular autocrine and paracrine communication (104–111). Different sets of purinergic receptors are expressed in the testis in a cell-specific manner, including Leydig cells (112, 113), Sertoli cells (108, 114), testicular peritubular cells (109), and pre- and post-meiotic germ cells (104, 115) (**Figure 3**). Several P2X receptors were initially described in the testis, including P2X1,2,3,5,7, while P2X4 and P2X6 were not detected in this organ (115). However, in a previous paper, *P2rx4* mRNA expression had been described in mouse seminiferous tubules (116). In later studies, P2X2, 4, 6, and 7 were detected in Leydig cells (103, 112), and electrophysiological experiments suggested that P2X2, 4, and 6 form heteromeric channels (112).

Regarding the roles of P1 receptors in the testis, the A1 receptor was first detected in rat testes (117, 118). In a separate study, adenosine receptors were detected in Sertoli cells by evaluating the binding of the adenosine analog cyclohexyladenosine in testicular cell extracts from immature rats (119). More recently, mRNA transcripts coding for all P1 receptors, A1, A2A, A2B, and A3, were observed in mouse testicular peritubular cells, while only A1, A2A, and A2B mRNAs were found in human peritubular cells (120). In the same study, A2B protein was localized by immunocytochemistry in human Sertoli cells, germ cells, and peritubular cells. In addition, adenosine and the A2B agonist BAY60-6583 increased IL6 and CXCL8 mRNA levels in isolated human testicular peritubular cells, showing a pro-inflammatory role for adenosine in the testis.

In Leydig and Sertoli cells purinergic signaling controls gonadotrophin responses (106, 107, 121–124). In Sertoli cells, ATP induced the release of calcium from intracellular stores, as well as the influx of calcium and sodium, indicating the participation of both P2X and P2Y receptors (105). ATP-induced sodium-dependent membrane depolarization in these cells triggered estradiol secretion (125). ATP also increased intracellular Ca^2+^ in Leydig cells triggering the secretion of testosterone. Moreover, in the mouse testis, Sertoli cells were shown to release ATP and adenosine, while germ cells and myoid peritubular cells could release adenosine (106, 122). The secretion of these extracellular mediators is under the control of follicle-stimulating hormone (FSH) and vitamin A (retinol), and participates in the paracrine regulation of testicular function (122, 126). Activation of both P2X and P2Y receptors by ATP in testicular peritubular cells induced their contractions, which triggered directional sperm movement within the mouse seminiferous tubules (127). Collectively, these studies showed that ATP participates in the autocrine regulation of Sertoli cells, as well as in a Sertoli cell to peritubular cell communication mechanism for the transport of spermatozoa in the testis.

Another example of testicular intercellular communication mediated by ATP was illustrated in an elegant study published by Fleck et al. in 2016 (104). Intimate interactions between Sertoli cells and the maturing germ cells are crucial for spermatogenesis. In this study, ATP secreted by Sertoli cells was proposed to activate P2X4 and P2X7 located in spermatogonial cells to modulate synchronized sperm development and release. Interestingly, ATP-induced ATP release was observed, indicating the presence of a mechanism that could increase the paracrine radius of locally generated signals. The testis is an immune-privileged organ whose luminal compartment is separated from the blood circulation by Sertoli cells that form the blood-testicular barrier. Thus, a local cell-cell intercellular communication network mediated by luminal factors such as ATP and adenosine would provide an ideal mechanism through which spermatogenesis could be performed in a synchronized manner while being protected from the peripheral immunological environment.

### Epididymis

At their exit from the testis, spermatozoa have acquired their overall morphology characterized by their long flagellum and head that contains densely packed DNA, but they remain immature cells. They acquire their fertilization capacity as they transit in the epididymis, a process called epididymal maturation (128–133). Another role of the epididymis is to protect sperm from the immune system while ensuring protection against ascending and blood pathogens (131, 134–138). Sperm maturation, protection, and storage occur in a unique epididymal luminal environment that has a high osmolarity, high potassium, low sodium, and low bicarbonate concentrations, and an acidic pH compared to blood (139–141). Communication networks between the different epithelial cell types that line the epididymal lumen, as well as between epithelial cells and spermatozoa, ensure tight regulation of this optimal environment (131, 142, 143). In addition, transcriptionally inactive spermatozoa acquire several new proteins that are essential for their fertilization ability as they transit along the epididymal tubule. This process occurs *via* the production of extracellular vesicles called epididymosomes, which are produced by epithelial cells and fuse with the sperm membrane (144–149).

In rodents, the epididymis is separated from the testis by the efferent ducts (150), and it is segmented into four main regions, the initial segment (IS), the caput, the corpus, and the cauda (151). In humans, efferent ducts occupy a large portion of the proximal epididymis, which is then divided into the caput, corpus, and cauda (152). To our knowledge, very little is known about purinergic signaling in the efferent ductules, except that the pro-inflammatory receptor P2Y14 is expressed in ciliated cells in the proximal regions and unidentified epithelial cells in the distal regions of the human efferent ducts (153). However, elaborate paracrine and autocrine purinergic signaling networks are present in the epididymis. The epididymal epithelium is lined by several cell types including narrow cells (located exclusively in the rodent IS), and clear cells (CC), principal cells (PC), and basal cells (BC).

As mentioned above, several purinergic receptors are located on the luminal side of transporting epithelia (4). The epididymis was the first intact epithelial organ where apical P2 receptors were shown to modulate transepithelial transport (154). Ten years later, the role of A1 and A2A receptors in the contractility of the cauda epididymis was demonstrated (155). Various purinergic receptors belonging to the P1, P2X, and P2Y families, and numerous ectonucleotidases are expressed in the epididymis in a cell type-specific and segment-specific manner (**Figure 3**) (7, 95, 98, 156–163).

**Figure 4:**
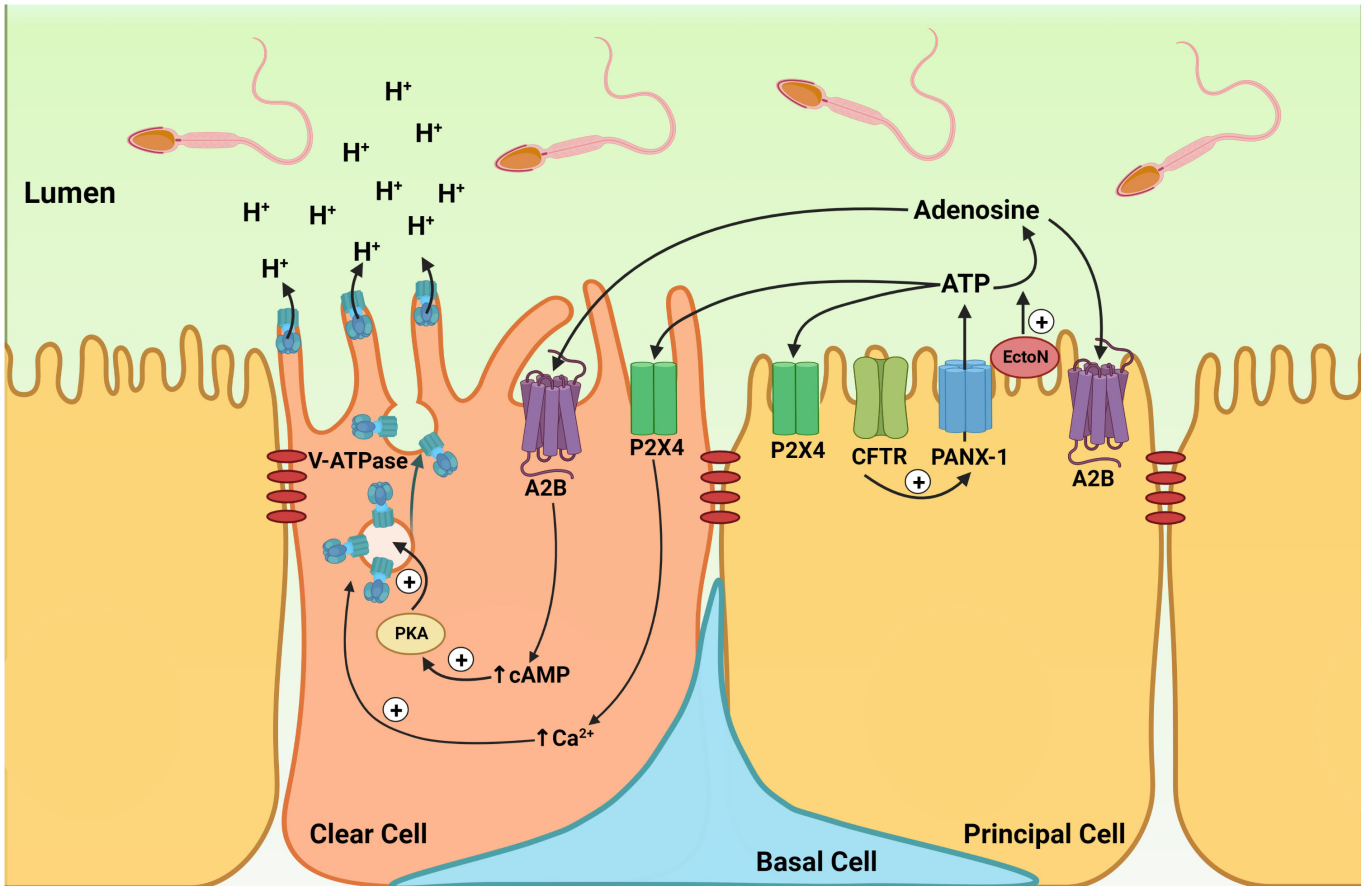
Cell-cell crosstalk model of purinergic regulation in the epididymis. Model showing the participation of purinergic regulation in the coordinated interaction between principal cells and clear cells, leading to the establishment of an optimal acidic luminal environment in the epididymis. Located on the apical membrane of PCs, CFTR activation stimulates ATP secretion by the pannexin-1 channel (PANX-1). Extracellular ATP is then hydrolyzed into adenosine by ectonucleotidases (EctoN) that are present on the apical surface of the epithelium. Luminal ATP activates the P2X4 receptor, and adenosine activates the A2B receptors located on the apical membrane of CC. Activation of A2B by adenosine leads to an elevation in intracellular cAMP. Activation of the cAMP/PKA pathway then induces V-ATPase apical membrane accumulation. Intracellular calcium also facilitates V-ATPase membrane accumulation in CCs, following P2X4 receptor activation by luminal ATP. The redistribution of V-ATPase from intracellular vesicles to the apical membrane results in the formation and elongation of V-ATPase-enriched microplicae in CCs, which stimulates proton secretion. The P2X4 and A2B receptors are also located on the apical membrane of PC, and their role in PC function is currently being evaluated.

In the cauda epididymis perfused *in vivo*, the addition of adenosine or ATP to the luminal perfusate induced the redistribution of the proton pumping V-ATPase from intracellular vesicles to the apical membrane in CC in rats (158) and mice (164) (**Figure 4**). Luminal acidification in this organ is essential for the maintenance of sperm in a quiescent state during their storage and favors the transfer of proteins from epithelial cells to spermatozoa *via* epididymosomes (140, 141, 148, 165–168). V-ATPase apical accumulation in CC depends upon elevations of intracellular cAMP and calcium (169–171). In the rat epididymis, the apical accumulation of V-ATPase elicited by luminal adenosine was inhibited by myristoylated protein kinase A inhibitor, indicating the contribution of either A2A or A2B receptors (158). Because only A1, A2B, and A3 were detected in epithelial cells isolated by laser-cut microdissection, and because A1 and A3 induce a decrease in cAMP, it was postulated that A2B was responsible for the adenosine action. This was further confirmed in the mouse epididymis, where A2B was located in the apical membrane of CC and where its specific agonist, BAY60-6583, induced V-ATPase apical accumulation in CC (164). A2B was also localized in the apical membrane of PC and its role in the regulation of these cells is currently being evaluated in our laboratory. V-ATPase-dependent proton secretion is activated at luminal alkaline pH (70, 164, 172). However, how these cells can sense and respond to variations in their extracellular environment remains incompletely understood. Modulation of purinergic signaling by extracellular pH would provide such a pH sensory mechanism. Indeed, some P2X receptors such as P2X4, as well as ectonucleotidases are activated at an alkaline pH (173–175). Increased hydrolysis of luminal ATP into adenosine by ectonucleotidases at alkaline pH versus acidic pH was shown to participate in V-ATPase dependent proton secretion (164). On the other hand, activation of P2X4 by ATP could provide an additional mechanism through which V-ATPase-dependent proton secretion would be activated at alkaline pH.

Significant ATP concentration was measured in the luminal perfusate collected in the vas deferens following luminal perfusion of the cauda epididymis indicating that epididymal epithelial cells secrete ATP (176). The cell line (DC2) representing epididymal PC (177) also secretes ATP (176). In the perfused cauda epididymis, the pannexin inhibitor carbenoxolone (CBX) partially prevented luminal pH recovery from an alkaline pH and inhibited alkaline pH-induced V-ATPase apical accumulation (164). Altogether, these results showed that ATP secretion by PC, followed by its rapid hydrolysis to produce adenosine, participates in the pH-induced activation of V-ATPase-dependent proton secretion by CC (164). This indicates the presence of a PC-CC crosstalk to activate proton secretion in CC (**Figure 4**). Interestingly, Cystic Fibrosis Transmembrane Regulator (CFTR), the gene mutated in cystic fibrosis also participates in ATP secretion by PC (176). Cystic fibrosis is associated with male infertility, and it will be interesting to determine whether dysfunction in ATP secretion might be involved in this medical condition.

**Figure 5:**
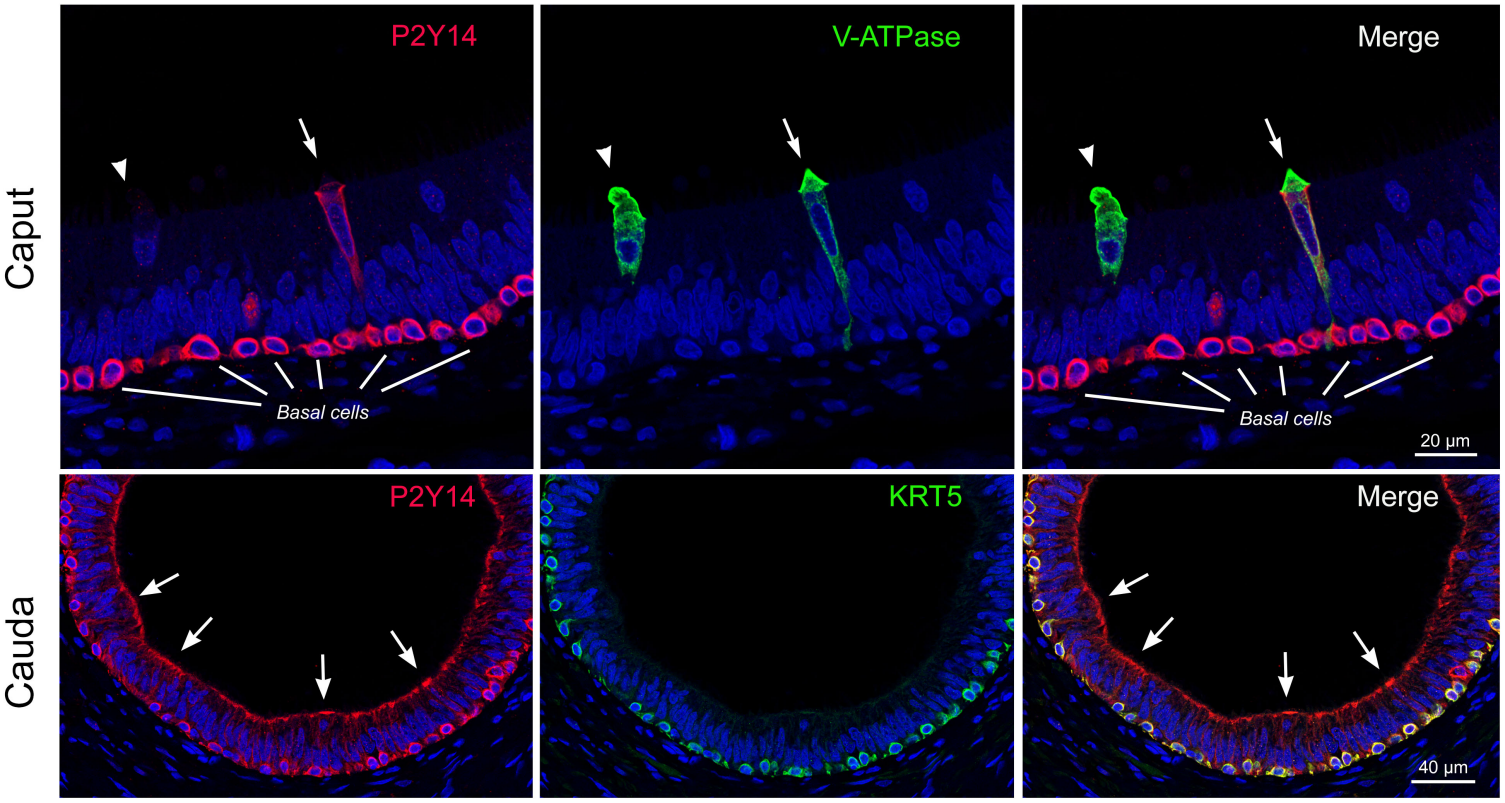
Localization of the pro-inflammatory P2Y14 receptor in the human epididymis. Top panels: Double immunofluorescence labeling for P2Y14 (red) and the B1 subunit of the V-ATPase (V-ATPase: green) in the human caput epididymis. Two V-ATPase-positive clear cells are shown (arrow and arrowhead). Only one clear cell is labeled for P2Y14 (arrow). Basal cells show strong labeling for P2Y14. Scale bar = 20 µm. Bottom panels: Double immunofluorescence labeling for P2Y14 (red) and cytokeratin 5 (KRT5: green) in the human cauda epididymis. Principal cells show strong apical labeling for P2Y14 (arrows). P2Y14 is also detected in KRT5-positive basal cells. Scale bar = 40 µm. Modified from Belardin et al. *Andrology* 2022.

In addition to its physiological role, purinergic signaling in the epididymis might also play a pro-inflammatory role. Epididymitis is a relatively common disorder compared to orchitis in outpatient clinics (178). ATP and UDP-glucose are DAMPs that could potentially trigger sterile inflammation by activating purinergic receptors in the epididymis. Pro-inflammatory purinergic signaling in the epididymis was recently indicated by the increase in CXCL10 expression induced in epithelial cells following exposure to damaged male germ cells (179). Although not investigated, damaged germ cells might release DAMPs, such as ATP and UDP-glucose, and activate purinergic receptors located on the apical membrane of epididymal epithelial cells. Interestingly, UDP-glucose which activates P2Y14 might participate in inflammatory disorders of the epididymis. In particular, accumulation of spermatozoa in the epididymis following vasectomy may induce congestive epididymitis and subsequent tissue structural and functional alterations. P2Y14 is expressed by BC in all epididymal regions of non-vasectomized men, as well as in PC located in the corpus and cauda regions (153) (**Figure 5**). A subpopulation of CC located in the caput region also showed strong P2Y14 labeling. In vasectomized men, an increase in *P2RY14* mRNA was observed in the corpus and cauda, and stronger apical labeling was detected in PC in the corpus region (153). Interestingly, the interstitium of the corpus and cauda epididymis of vasectomized men was characterized by the presence of numerous CD45^+^ leukocytes, compared to non-vasectomized men. Several CD45^+^ leukocytes were also observed in the corpus lumen following a vasectomy, where they appeared to have internalized spermatozoa. Thus, this study highlighted the potential role of P2Y14 in the generation of an inflamed-prone environment in the epididymis following vasectomy. In addition, UDP-glucose is involved in the homeostasis of the bacterial envelope, and bacteria themselves could modulate ATP secretion by epithelial cells (33, 72). Future studies will be required to test the role of purinergic signaling in pathogen-induced epididymitis.

In spermatozoa isolated from the mouse epididymis, P2X2 participates in an ATP-mediated intracellular Ca^2+^ increase (180). While spermatozoa from *P2rx2* KO mice have normal functional parameters, the fertility of *P2rx2* ^-/-^ males rapidly declines with frequent mating. It was suggested that P2X2 confers a selection advantage by enhancing the continuous production of functional sperm under conditions of high demand. ATP is also involved in acrosomal exocytosis, a process that releases enzymes essential for sperm penetration into the egg (181).

### Vas deferens

While several purinergic receptors are located in the vas deferens (**Figure 3**), purinergic signaling in this tissue is best known for its action on smooth muscle contraction and semen quality, which has been demonstrated in several species including humans (93, 95, 97, 182–185). Vas deferens smooth muscle contraction is initiated by motor nerve stimulation and is mediated by ATP and noradrenaline, which act as co-transmitters of the motor response in several species (185–187). In rats, guinea pigs, and rabbits, vas deferens muscle contraction is biphasic, and ATP acts through P2X receptors to stimulate the initial fast phase of contraction (186, 188–190). In later studies, ATP was shown to play opposite roles through inhibition of noradrenaline release mediated by P2Y12 or P2Y13 receptors, and the increase in noradrenaline release mediated by homomeric P2X1, P2X3 or heteromeric P2X2/P2X3 receptors (187). Confirmation of the role of the P2X1 receptor located on the smooth muscle membrane in the contractile response elicited by ATP was demonstrated in several additional studies (95, 183, 184). In support of this, null-mice for the P2X1 receptor have a 90% fertility reduction caused by reduced nerve-induced vas deferens smooth muscle contraction (95). Of note, no effect on spermatozoa was observed in these mice.

In addition to ATP, adenosine is also a key mediator of smooth muscle contraction in the vas deferens (93) and the epididymis (191). The A1 and A2 receptors were shown to enhance or inhibit contractions, respectively (187, 192, 193). A2B inhibited contractions in both the prostatic and epididymal regions of the vas deferens, while A2A was involved in the prostatic region only (193, 194). Inhibition of contractility by A2B was later shown to be mediated *via* the release of noradrenaline and activation of potassium channels, which involved the participation of both protein kinase A and G (195).

Regarding the purinergic modulation of transepithelial transport in the vas deferens, luminal but not basolateral adenosine stimulates anion secretion in cultured epithelial cells isolated from porcine and human vas deferens (196, 197). Adenosine also rapidly increased anion secretion in freshly excised human vas deferens (196). ATPγS, the non-hydrolysable form of ATP, was without effect, excluding the role of ATP receptors in the modulation of anion transport. Significant increase of cAMP was elicited by luminal adenosine indicating the role of A2A or A2B receptors (196). Extensive purinergic signaling that operates on the luminal side of the epithelium was proposed to modulate the luminal environment in which spermatozoa transit during ejaculation. Similar to the epididymis, adenosine may be produced in the vas deferens following ATP secretion and hydrolysis by ectonucleotidases. Of note, in the kidney ATP secretion is activated by flow (33), and it is possible that bicarbonate secretion would be increased in the vas deferens following ejaculation-induced flow. Bicarbonate induces a cAMP increase in spermatozoa through direct activation of bicarbonate-activated adenylyl cyclase (sAC; ADCY10) (198). This process is essential to initiate sperm motility and capacitation. Thus, activation of bicarbonate secretion in the vas deferens would help prime spermatozoa while they start their long journey to reach and fertilize the egg. Finally, P2Y14 is expressed in BC and in the apical membrane of PC in the human vas deferens, indicating its potential role in immune responses triggered by the DAMP molecule UDP-glucose in this organ (153).

## Conclusion and perspective

Infertility is a major public health issue that affects nearly 1 in 7 couples in North America, and male infertility is the culprit in up to half of these infertility cases (199, 200). Importantly, 40-50% of male infertility is still labeled idiopathic (201), illustrating our incomplete knowledge about male reproduction health. Male infertility is caused by defective spermatogenesis and/or by sperm that have reduced fertilization functions, and the establishment of male reproductive health depends on several physiological processes that take place in the testis and the post-testicular reproductive tract. Purinergic signaling plays a variety of physiological and pathophysiological roles in the male reproductive system, but our knowledge in this context remains limited. While several purinergic receptors are expressed in the testicular and post-testicular tract, only P2X1 was shown to be indispensable for the establishment of male fertility. The vast array of purinergic receptors located all along the male reproductive tract could certainly provide several compensatory mechanisms, which would mask the crucial role of each receptor. When and how these individual receptors become dysfunctional and drive specific disease processes in the male reproductive tract is just starting to be elucidated. Future progress in our understanding of purinergic signaling in male reproduction will depend on increasing information regarding the molecular structures of P1, P2Y, and P2X receptors, the generation of novel knock-out mice, and the development of specific receptor agonists and antagonists.

## Author contributions

LB: Drafting and revising the manuscript; and final approval of the manuscript to be submitted for publication. KB: Drafting and revising the manuscript; and final approval of the manuscript to be submitted for publication. CL: Revising the manuscript; and final approval of the manuscript to be submitted for publication. MB: Revising the manuscript; and final approval of the manuscript to be submitted for publication. SB: Writing and revising the manuscript; and final approval of the version to be submitted for publication. All authors contributed to the article and approved the submitted version.

## Funding

This study was supported by the Canada Research Chair in Epithelial Dynamics of Kidney and Reproductive Organs (to SB), the Foundation of the Centre Hospitalier Universitaire de Québec (to SB), NIH grant HD040793 (to SB), by Canadian Institutes of Health Research (CIHR) grant 183711(to. SB), by the Fonds de Recherche du Québec en Santé (FRQS) (to LB.) and by NIH grant HD104672-01 (to MB).

## Conflict of interest

S.B. is the scientific co-founder of Kantum Pharma.

The remaining authors declare that the research was conducted in the absence of any commercial or financial relationships that could be construed as a potential conflict of interest.

## Publisher’s note

All claims expressed in this article are solely those of the authors and do not necessarily represent those of their affiliated organizations, or those of the publisher, the editors and the reviewers. Any product that may be evaluated in this article, or claim that may be made by its manufacturer, is not guaranteed or endorsed by the publisher.
